# Effect of Ayurveda and Siddha interventions in the management of chronic lymphocytic leukemia: A case report

**DOI:** 10.1016/j.jaim.2024.101017

**Published:** 2024-12-14

**Authors:** Abhay Kumar Prajapati, Parvathy Venate, T. Monika, V.G. Huddar

**Affiliations:** a Department of Kayachikitsa, All India Institute of Ayurveda, New Delhi, 110076, India; b National Institute of Siddha Chennai, Tamil Nadu, 600047, India

**Keywords:** CLL ayurveda, Leukemia, CAM, Siddha, Rajayakshma, AYUSH

## Abstract

Chronic Lymphocytic Leukemia (CLL) is a hematological malignancy marked by the clonal proliferation of the mature, yet dysfunctional B lymphocytes in blood and its subsequent infiltration of the marrow, lymphoid tissues and spleen. The resultant immune dysfunction exposes the body to multiple infections and is a cause of major mortality. Here, we discuss the case of a 46-year-old male suffering from CLL who presented with long-standing episodes of low-grade fever, productive cough and throat irritation, lethargy and pedal edema. He was treatment-naïve at the time of presentation. He was treated in lines of Rajayakshma as per Ayurveda, and *Kuruthiputru* as per Siddha medicine. After three years of treatment with continued follow up till date, comprising of *Shamana* C*hikitsa* (Ayurveda and Siddha internal medications), *Sodhana* (bio purificatory methods)through *Mridu Virechana* (therapeutic purgation) *using Trivrut* A*valeha* 35g, Guduchi *Rasayana (Tinospora cordifolia)* and *Yashtimadhu Rasayana(Glycyrrhiza glabra),* patient reported reduction in frequency of infections, improved performance score with a mean reduction in TLC counts by 100 thousand/cu. mm in 10 months starting intervention. Presently the patient is stable with occasional episodes of dry cough and generalized weakness.

Integrated practices involving Ayurveda and Siddha medicines may be beneficial to patients suffering from CLL by reducing the episodes of infections, improving the quality of life while pacifying the deranged hematological parameters to certain extent.

## 1 Introduction

Chronic lymphocytic leukemia (CLL) is the most common type of adult leukemia in the developed countries. It is characterized by the clonal proliferation and accumulation of mature, yet dysfunctional B-cells within the blood, bone marrow, lymph nodes, and spleen [1]. This intrinsic state of immune dysregulation compounded by the chemo-induced immunosuppression predisposes the patient to a plethora of infections contributing to 30–50% of mortality in CLL [[2], [3], [4]]. The clinical course takes two distinct phases-an initial indolent phase requiring close follow-up, eventually transforming into a progressive disease requiring immediate therapy. Symptoms includes lethargy, easy fatiguability, night sweats, painless lymphadenopathy, weight loss, body aches and recurrent opportunistic infections. The diagnosis is through peripheral blood smear (≥5 × 10^9^/L monoclonal B lymphocytes in the peripheral blood) and confirmed by flow cytometry and immunophenotyping of peripheral blood. Rai and Binet staging systems are used for risk stratification and interventional decisions ^[^[5]^.^ [6]^].^

In spite of advances in the treatment, CLL remains an incurable disease. The goals of therapy are to improve quality of life (QoL) and to prolong survival. The standard treatment in early asymptomatic disease is to watch-and-wait [7]. The treatment is started only in the symptomatic patients (fulfilling the 2018 iwCLL criteria) and comprises of oral targeted therapy (BTKi and venetoclax), immunotherapy and hemopoetic stem cell transplant [8]. With the advent of targeted therapy, there is improved efficacy and tolerability to the treatment, yet lifelong dependency to medicine poses a major threat to compliance.

In Ayurveda, the clinical trajectory of CLL traverses through multiple *Dhatu-pradoshaja vikaras* such as *Vishama Jwara, Pandu, Raktapitta, Asthi-majjagata* *Vata* and culminates into the pathogenesis of *Rajayakshma*. *Jatharagnimandya* results in the formation of *Vikruta Rasa dhatu* and manifests as *Kasa* (bouts of cough), *Amsatapa* (shoulder pain), *Jwara* (fever), *Parswasoola* (chest pain), *Kapha*-*Rakta Chardi* (hemoptysis), *Swasa* (dyspnea), *Varcogada* (diarrhea) and *Aruchi* (anorexia). As the disease progresses, all the subsequent dhatus and ultimately *ojas* gets depleted and the patient succumbs [9]. The mainstay treatment includes *Shamana Chikitsa* (pacificatory medicines) incorporating drugs that are *Agnideepana* (enhance digestion), *Balya* (improves strength), *Srotosuddhikara* (removes metabolic by-products) and Rasayana (rejuvenative). If patient is fit for Panchakarma, *Snehapana* (internal oleation), *Mridu Shodhana* (mild bio-purificatory procedures) by means of *Vamana* (emesis therapy) and *Snigdha Virechana* (medicated purgation) may be done, followed by appropriate *Rasayana* [10].

As per the Siddha literature evidence, Chronic Lymphoid Leukemia (CLL) is correlated with *Ratha vipuruthi* caused by the imbalance in the *Udal Thattukal* (physical constituents) - *Seneer* (blood), *Enbu* (bone) and *Moolai* (marrow) [11]. It is characterized by *Suram* (Fever), *Illaippu & Ellai*, (respiratory infections), *Vaiyuru uppusam* (abdominal distension), *Udal illaithal* (emaciation), *Nina Neer Mudichu* (swollen lymph nodes), *Seriyamai* (indigestion), *Pasi inamai* (loss of appetite), *Paandu* (anemia), *Vaai Kumatal* (nausea) and *Thalaisuthal* (giddiness).

### 1.1 Patient's information

A 46-year-old male, diagnosed case of CLL, presented at Integrated cancer OPD of AIIA, New Delhi on Feb 28, 2021 with complaints of frequent episodes of low-grade fever associated with productive cough, throat irritation, easy fatiguability, pain and swelling over both legs aggravated since past one year. Diagnosed in 2017, he had been treatment naive at the time of presentation.

**Family History**- No known history of malignancies among his first and second-degree relatives.

**Personal history**- Patient partakes mixed diet, is constipated, has normal bladder movements as well as normal sleep pattern. He was addicted to tobacco chewing for 22 years but reformed since the past 5 years.

### 1.2 Clinical findings

He is of lean built (wt-46 kg), malnourished with BMI of 18.68 Kg/m^2^. Pallor-absent with painless mobile lymphadenopathy at multiple sites- Cervical, Axillary, Inguinal and Abdominal lymph nodes. There was bilateral pedal edema-pitting type with spontaneous resolution on lying down. Icterus, clubbing and cyanosis-absent. Abdomen was soft, non-tender with no palpable hepato-splenomegaly. Cardiovascular and respiratory system examinations were normal.

The patient was of *V*ata-*P*itta *P*rakriti, and had *Vishamagni* (irregular digestive power), *Krura Koshta* (nature of bowel), *Madhya* (moderate) *Vaya* (age), *Satva bala* (will) and *Avara Aharashakti* (strength of digestion), *Sara* (quality of body tissues), *Satmya* (compatible), *Pramana* (body stature), *Samhanana* (compactness of body) and *Vyayama shakti* (physical endurance).

The *Nidana* (causative factors) suggestive of longstanding V*ata-*P*rakopa* could be traced in the patient owing to his occupation as a driver like *akala-pramita bhojana*(untimely food intake in less quantity), *Vega Dharana*(suppressing natural urges)and *Ratri Jagarana(*sleep deprivation*)*. The excess use of tobacco may also have contributed as a *Vikashi Dravya* by destroying the compactness of the bodily tissues. With history of Pulmonary TB, there is already a *vaigunya* at *pranavaha srotas*. The resultant *Vata-pradhana Sannipata Kopa* happening in the *Abhyantara Rogamarga* at *Amasaya* extends into the *Rasavaha*, *Raktavaha* and *Pranavaha srotases* resulting in the manifestation as *Rajayakshma*. The disease being *Jeerna* (beyond one year after onset) with extensions into the *Madhyama rogamarga* (*asthi* -*majja*) and associated with *bala-mamsa kshaya* (weight loss) is to be understood as difficult to cure.

### 1.3 Timeline

Patient had a past history of Pulmonary Tuberculosis in 1989, treated with nine months of Anti-tubercular treatment (ATT) (documents not available). In March 2017, following a treatment refractory neck swelling associated with persistent leukocytosis, he was evaluated at AIIMS, New Delhi and diagnosed of having CLL (Rai II, Binet B). For the next two years, he was kept on regular follow-up. There were six episodes of clinically recorded respiratory infections managed through antibiotics, and two instances of fungal infection over the skin during the period. In May 2019 he developed multiple episodes of frank haemoptysis (BAL negative for AFB and fungal growth) and thrombocytopenia (87000/cu.mm). Bone marrow cytology revealed near total replacement by monomorphic lymphocytes and the patient was upstaged as Rai IV, Binet C and planned for Chemo-immunotherapy (CIT). Due to financial constraints, he defaulted further treatment. The frequency of the respiratory infections increased with each febrile episode lasting more than 20 days a month. It was associated with yellowish sputum, often accompanied by streaky hemoptysis and severe bony pains. Anorexia ensued, followed by constipation and significant weight loss of 24 kg in 2 years. He had to give up working and had difficulty in carrying out self-care. By November 2020, he developed intractable pedal edema bilaterally, further restricting him to bed. Following a friend's suggestion, he approached AIIA for possible alternate solutions.

### 1.4 Diagnostic assessment

The diagnostic work-up was done at AIIMS in March 2017 and continued till June 2019. The peripheral smear showed smudge cells. The flow cytometry revealed the presence of monoclonal B-cell population that expressed CD19, CD20, CD5, CD23, CD200 and light chain restriction for lambda. FISH was positive for Deletion 13q14.3 in 90% and (14q32) IGHV gene rearrangements in 86% cells. CECT Thorax and Abdomen revealed hepatomegaly (18 cm), splenomegaly (14.8 cm) and bilateral axillary lymphadenopathy.

Based on the initial work-up, he was characterized as Rai II and Binet B staging. Rai II staging is characterized by lymphocytosis in blood and marrow with hepatomegaly and/or splenomegaly, with or without lymphadenopathy [5]. Binet B clinical staging is characterized by enlargement of ≥3 lymphoid areas, no anemia or thrombocytopenia [6]. The median survival for the patient's staging was approximately 6 years [5,6]. Being asymptomatic, he was planned for observation and regular follow-up.

In 2019, he developed multiple episodes of hemoptysis (>10 ml, red-colored clots). Bronchoalveolar Lavage was negative for AFB and fungal growth. Radiological imaging through CECT Thorax and Abdomen showed ground-glass opacities in the left upper lobe, splenomegaly and bilateral axillary lymphadenopathy. BMA study revealed near total replacement by monomorphic lymphocytes with 3% constituted by prolymphocytes. Marrow infiltration by these cells were also observed. Thrombocytopenia (87000/cu.mm) set in and the patient was restaged as Rai IV, Binet C with an expected median survival of less than 2 years [5,6]. During the course of treatment at AIIA, hemogram was repeated periodically and imaging was done when deemed necessary. (A tabular representation of the same has been provided in Table 1).

**Table 1:** Timeline of investigations.

Date of Investigation	Hb (gm/dl)	TLC (Thousands/cc)	Platelets (Thou/mm^3^)	ESR (mm/1stHr)
02-11-2016	13.4	56.5	128	
25-02-2017	12.9	40.3	166	29
March 07, 2017	Peripheral smear Leukocytosis with numerous smudge cells seen. 1 NCNC RBCs seen.			
March 14, 2017	Flow- cytometry Suggestive of Chronic Lymphocytic Leukemia with Matutes Score - 5/5			
05-04-2017	13.6	88.2	104	
April 24, 2017	CECT Thorax & Abdomen Aspirate from right cervical swelling shows reactive lymphoid tissues.			
03-07-2017	13.8	123.7	107	
04-09-2017	15	89.6	80	
02-10-2017	13.7	85.5	90	
October 16, 2017	FISH Positive for Deletion 13q14.3 in 90% and (14q32) IGHV gene rearrangements in 86% cells			
08-04-2017	13.2	82.1	100	10
04-09-2017	15	89.6	130	
25-01-2018	14.4	138.6	64	
08-04-2018	11	88.6	110	
29-06-2018	12.2	188.6	120	
29-09-2018	11.4	199	74	
October 07, 2018	RT-PCR (IgVH mutation) - Hypermutation >2%			
09-10-2018	11.4	199	120	
March 15, 2019	BMA & Biopsy Bone marrow is cellular, shows near total replacement by monomorphic lymphocytes. Prolymphocytes constitute 3%. Bone marrow biopsy is adequate and hypercellular, shows interstitial and diffuse infiltration by monomorphic lymphoid cells.			
26-04-2019	11.7	203	112	
27-05-2019	11.1	252	110	–
June 02, 2019	CECT Thorax & Abdomen GGO in left upper lobe. Enlarged bilateral axillary lymphadenopathy (largest 13.5 × 18.5 mm). Splenomegaly			
June 11, 2019	BAL for AFB, Culture BAL shows alveolar macrophages, columnar cells. AFB negative.			
29-07-2019	11.2	232	68	
27-02-2021	13.2	282	133	27
19-10-2021	11.9	300.7	137	40
29-12-2021	10.9	200.7	119	55
29-03-2022	10.8	199.99	130	38
29-04-2022	11	179	123	24
06-09-2022	12.1	142	130	–
September 06, 2022	USG abdomen Splenomegaly 15.7 cm was present			
20-10-2022	12.2	193.6	107	26
14-12-2022	12.1	169.7	143	38
03-03-2023	12.5	190	112	11
05-04-2023	12.2	168.69	119	09
14-06-2023	12.0	150.1	185	13
14-12-2023	12.3	150	104	7
20-03-2024	11.3	142.9	100	9
08-05-2024	12.2	168	127	132

### 1.5 Therapeutic intervention

The condition was assessed as *Vata*-*pradhana sannipata dushti* involving all dhatus, predominantly *Rasavaha, Raktavaha, Asthivaha* and *Majjavaha srotasas* (Refer Table 2, Table 3 for treatment details with rationale).

**Table 2:** Details of treatment given.

INTENT	MEDICINES & PROCEDURES	Duration of Treatment (month wise)													
		Feb 21-Aug21	Sept 21	Oct21-Dec21	Jan21-Mar22	Apr 22	May22-June22	Jun22-Aug 22	Sept 2022	Sept 22- Nov 2022	Sep 22-Oct 22	Nov 22- Jan 23	Feb 23	Mar 23-Apr 23	Apr 23- May 23
SHAMANA CHIKITSA	*Tab Rasayan churna vati* 1 BD	✓			✓										
	*Sitopaladi churna (*2 gm*) + Yashtimadhu churna (*1 gm*) + Tankana Bhasma(*250 mg*) BD*	✓										✓			
	*Tab Punarnava Mandoora* 1 BD	✓		✓	✓										
	*Panchatikta Guggulu Ghrita* 10 ml *BD*	✓													
	*Syp Madhulai Manappagu* 5ml BD	✓					✓	✓							✓
	*Amukkara Choornam* 2g BD	✓													✓
	*Tab Styplon 1TDS*			✓											
	*Tab Chandrakala ras TDS*			✓	✓		✓	✓							
	*Amalaki churna (*2 gm*) + Yashtimadhu churna(*1 gm*)* + *Mukta pishti(*250mg*) BD*			✓											
	*Seenthil Choornam 5g BD*			✓											✓
	*Brihat. Manjishthadi Kashayam*				✓					✓					
	*Nagakesara Churna(*3 gm*) + Guduchi Sattva(*250 mg*) + Swarna gairika (*250mg*)* + *KamadudhaRas(*125mg*) BD*				✓		✓	✓							
	*Nellikai legiyam-* 3 gm *BD*				✓										
	*Tab. Bhavana Kadukai* 1 BD				✓										
	*Panchatikta Guggulu Ghrita -*10 ml *BD*						✓	✓			✓				
	*Syp M Vasaco -*10 ml *BD*						✓					✓			
	*Mahavallathi Legiyam* 3 gm *OD*						✓	✓		✓					
	*Drakshadi Churnam* 3 gm *BD*						✓								
	*Patolakaturohinyaadi Kashaayam* 30 ml *BD*									✓	✓	✓		✓	✓
	*Saribadyasava + Lohasava-* 20ml *BD*									✓					
	*Samshamani vati (*500 mg*)* + *64 Prahari pippali(*250 mg*) + Nagakeshar churna (*3 gm*) + Yashtimadhu Churna(*3 gm*) BD*									✓	✓	✓			
	*Avipattikar Churna* 6 gm *HS*										✓	✓		✓	
	*Tab Dhatri Lauha 1 BD*										✓				
	*Kushmaanda Rasayana* 10 gm *BD*										✓				
	*Tab M Liv 1 BD*											✓		✓	✓
	*Talishadi churna (*2 gm*)* + *Vasadi churna (*2 gm*)* + *Yashtimadhu Churna(*1 gm*) BD*										✓	✓		✓	✓
	*Tab Shringarabhra Rasa* 1 *BD*													✓	✓
	*Agastya Rasayana* 10 gm *BD*														✓
SODHANA	*-****Snehapana****-* 5 days *Panchatikta guggulu ghrita* (30ml–170ml) *-****Sarvanga Abhyanga****-* 3 days *Chandana-bala-lakshadi taila* *-****Sarvanga bashpasweda****-* 3 days *Dasamoola kwath* *-****Virechana****-* 1 day *Trivrut avaleha 35g* + *lukewarm milk* 100ml Number of vegas- 13								✓						
RASAYANA	***Guduchi Rasayana (Cap. Guduchi-* 500 mg*)*** D1- 4 capsules D2- 6 capsules D3- 8 capsules D4- 10 capsules D5-D30 - 12 capsules		✓												
	***Yashtimadhu Rasayana-Yashtimadhu churna****(YC****) + Guduchi satwa****(GS****)*** D1-D4- *YC*(8g) + *GS* (2g) D5-D8- *YC* (15g) + *GS* (5g) D9-D12- *YC* (25g) + *GS* (5g) D13-D30- *YC* (35g) + *GS* (5g)					✓							✓

**Table 3:** Treatment given with rationale.

Lakshanas (symptoms)	Samprapti (pathology)	Chikitsa Siddhanta (Rationale)	Ayurvedic/Siddha formulations
Productive cough (*kasa*), throat irritation (*kanthodhwamsa*), running nose (*pratisyaya*), heaviness of head (*siro-gurutwa*)	*Kapha-vata*	*Kaphacchedana, vatanulomana*	***Churna****- Taleesadi, Sitopaladi, Madhuyashti, Tankan Bhasma* ***Vati****- Khadiradi, Lavangadi* ***Avaleha****- Agastya Rasayana, Kooshmanda Rasayana, Mahavallathi legiyam* ***Liquids****- Syp. M- Vasako*
Low- grade fever with cough	*Kapha-vata jwara*	*Pachana*	*Laxmivilas ras* *Samshamani vati* ***Siddha****- Seenthil churna*
Pedal edema, lymphadenopathy (*sopha*)	*kapha*	*Kapha-pitta samana, rakta prasadana*	*Brihat manjishtadi kashayam, Patolakaturohinyadi kashayam,* *Punarnava Mandoora*
Hemoptysis (*sonitha stheevana)*	*Pitta-vata prakopa, kapha kshaya (avalambaka)*	*Pitta shamana* *Rakta sthambhana* *Balya*	***Tablets****- Chandrakala ras, Kamadudha ras, Mukta pishti,* *T. STYPLON* ***Churna****- Amalaki, Yashtimadhu, Nagakesara* ***Kwath****- Patolakaturohinyadi* *Brihat Manjishtadi*
Reduced appetite (*aruchi*), bloating (*atopa*), Constipation (*vibandha*)	*Kapha-vata*	*Amapachana* *Apana vata anulomana*	*Avipattikara churna* *T. M-LIV* ***Siddha****- Bhavana Kadukkai*
Lethargy (*tandra*), Emaciation (*bala-mamsa kshaya*), Muscle cramps (*soola*)	*Vata*	*Srotosodhana* *Vata-samana* *Balya* *Rasayana*	*Panchatikta guggulu ghrita* *Dhatri lauha* *Rasayana churna(Guduchi, Amalaki, Gokshura)* *Guduchi Rasayana (Vardhamana)* ***Siddha****- Nellikai legiyam* *Drakshathi churna* *Amukkura churna (W. sominifera)*

### 1.6 Follow-up and outcomes

The patient has been regularly followed-up every month with Complete hemogram for assessment. In the first-follow up after 30 days, the pedal edema and muscle cramps were completely relieved. Low grade fever had resolved, however, febrile episodes accompanied by productive cough recurs every three months, none requiring hospitalization. After 761 days of follow-up, the performance score of the patient has improved from ECOG PS 3 to 1. There is remarkable weight gain from 46kg to 54 kg. TLC dropped from 282 thousand/cu. mm to 168 thousand/cu. mm. There were no instances of thrombocytopenia or serious infections (Grade 3–4 CTCAE). The patient reports a significant improvement in the quality of life. He was compliant to the treatment and experienced no major side-effects.

## 2 Discussion

Recurrent infections are the major cause for morbidity and mortality in Chronic lymphocytic leukemia contributing to 30–50% of the deaths. This susceptibility to infections is multifactorial-inherent immune deficits (hypogammaglobulinemia, defects in T-cells, suppressor natural killer cells, neutrophils, dendritic cells and complement system) as well as treatment induced (chemo-induced myelosuppression, mucosal barrier disruption and T-cell suppression) [12,13]. The disease is incurable warranting life-long medications that equates to diminished QoL, financial burden and poor compliance [14,15].

In this case, the interdisciplinary approach through Ayurveda and Siddha medicines have improved both clinical and hematological parameters and conferred good palliation to the patient.

The pathogenesis of CLL revolves around vitiation of rasa and rakta dhatus. In most cases, as the disease progresses, the subsequent dhatus undergo faulty dhatuparinama (formation of dhatus) and clinical presentation changes to *sosa* (phthisis).

In this case, the patient presented with a plethora of symptoms afflicting the *Pranavaha, Rasavaha, Asthivaha* and *Majjavaha srotases*. Hence, the treatment has to be multifaceted-providing symptomatic relief, improve general health, prevent further progression of the disease. The three arms of treatment- *Shodhana*, *Shamana* and *Rasayana* were employed giving due consideration to the strength of the patient and the extent of vitiation of the dosas. Formulations like *Sitopaladi churna, Madhuyasti churna, Tankan Bhasma, Taleesadi churna, Khadiradi vati* helps in *kaphacchedana*. *Agastya Rasayana, Kushmanda Rasayana* and *Mahavallathi legiyam* pacifies the deranged vata and improves the functioning of the *pranavaha srotas*. *Patolakaturohinyadi kwath, Brihat Manjishtadi kwath* and *Punarnava mandoora* was intended to correct *rasa dhatwagni* and aid in proper *dhatu parinama*. *Panchatikta Guggulu ghrita* is *srotosodhana* and *balya* and its indication-*Asthi- Majjagata vata, Yakshma, Arbuda* may be of benefit to this patient. *Rasayana churna* and *Madhulai manappagu* replenishes the *Rasa* and *Amukkura* (*W.somnifera*) rejuvenates and improves body strength.

To prevent further progression, *Rasayana Chikitsa* was planned. *Guduchi* was chosen for Rasayana owing to the *Tridosha-hara* property and action at the level of *Rasa-rakta dhatus*. It is known to possess potent anti-oxidant, anti-cancerous and immune-modulatory effects [[16], [17], [18]].

In the course of the treatment, the patient developed multiple episodes of hemoptysis (3–5 episodes for a week, 10–20 ml, clots present) with no systemic symptoms. *Sonita stheevana* due to *Pitta-Kapha prakopa* was identified and *Raktapittahara Chikitsa* was started. The primary concern was to arrest the bleeding. The medications prescribed like T. Styplon (*Amla, Sariva, Lodhra, Pravala), Chandrakala ras, Mukta pishti, Madhuyasti churna* were predominantly *Tikta-madhura* in *rasa, Sheeta Virya, Snigdha and Stambhana* qualities.

In the late spring of 2022, patient again presented with hemoptysis (2 episodes, <10 ml, mixed with sputum). *Yashtimadhu Rasayana* was started. *Yashtimadhu* possess antitussive, anti-inflammatory, immune-stimulatory effects and exhibits anti-proliferative effects against leukemic cells [[19], [20], [21], [22]]. The regimen comprising of an escalating dose of *Yashtimadhu churna* (maximum up to 35 g) along with *Guduchi satwa* (up to 5 g) was done for 30 days. The patient experienced abdominal discomfort during the last 4 days, associated with reduced appetite. But the hemoptysis completely resolved.

Bio purificatory procedures like virechana are of utmost importance in the management of chronic conditions like cancer. When the patient attained adequate body strength, virechana was planned in the early autumn (season of pitta vitiation). Oral medications were continued as per the symptoms. The active ingredients in the formulations like Am*alaki* [23]*, Bhallataka* [24]*, Dadima* [25]*, Draksha* [26] and *Aswagandha* [27] possess antineoplastic activity in leukemic cells.

The patient is continuing treatment with us more than three years now and reports significant improvement in his weight (54 kg now from 46 kg), energy levels (ECOG PS1), fewer bouts of fever and productive cough (fortnightly to once in 2–3 months now). The hematological parameters are also encouraging with reduction in TLC count from 282 to 168 thousand per cu. mm, the Hb and platelet counts remain stable. Similar findings have been reported by G Haskin et al. through the use of supplements and alternative medications in managing indolent case of CLL in elderly [28].

## 3 Conclusion

Integrative approach using Ayurveda and Siddha medicines is beneficial in the palliation of the Chronic lymphocytic leukemia by stabilizing the deranged hematological parameters including symptoms, reducing susceptibility to infections, enhancing the strength of the body and metabolism and thereby overall quality of the life. The patient had good compliance to the intervention and there were no adverse reactions seen during the period of intervention. Thus, traditional systems of medicine can offer safe and effective treatment without causing much economic burden.

## Patient perspective

As of June 2024, the patient feels remarkable improvement in this general health and well-being. The frequency and intensity of cough and hemoptysis has reduced, and pedal edema completely resolved. In his words, *“*I was unable to stand in the queue to get OP ticket on the first day of my visit and had to seek help from kind-hearted attendants. Now I walk 3–4 km every day and can carry out all my routine activities without assistance''.

## Informed consent

The article is being published with written consent from the patient.

## Sources of funding

The work was funded byAll India Institute of Ayurveda, New Delhi.

## Author Contributions

AKP- collection of data and preparation of original draft; PV- review andrevise draft; TM- review and revise draft; VGH- conceptualisation, supervision and review of draft.

## Declaration of generative AI in scientific writing

The authors declare that we have not used generative AI and AI-assisted technologies in the writing process.

## Conflict of interest

The authors declare that they have no known competing financial interests or personal relationships that could have appeared to influence the work reported in this paper.

## References

[1] Hallek M., Cheson B.D., Catovsky D. (2018). iwCLL guidelines for diagnosis, indications for treatment, response assessment, and supportive management of CLL. Blood.

[2] Forconi F., Moss P. (2015 Jul 30). Perturbation of the normal immune system in patients with CLL. Blood.

[3] Dhalla F., Lucas M., Schuh A., Bhole M., Jain R., Patel S.Y., Misbah S., Chapel H. (2014). Antibody deficiency secondary to chronic lymphocytic leukemia: should patients be treated with prophylactic replacement immunoglobulin?. J Clin Immunol.

[4] Kipps T., Stevenson F., Wu C. (2017). Chronic lymphocytic leukaemia. Nat Rev Dis Primers.

[5] Rai K.R., Sawitsky A., Cronkite E.P., Chanana A.D., Levy R.N., Pasternack B.S. (1975). Clinical staging of chronic lymphocytic leukemia. Blood.

[6] Binet JL, Auquier A, Dighiero G, Chastang C, Piguet H, Goasguen J (1981). A new prognostic classification of chronic lymphocytic leukemia derived from a multivariate survival analysis. Cancer.

[7] Eichhorst B, Robak T, Montserrat E, Ghia P, Niemann CU, Kater AP (2021). ESMO Clinical Practice Guidelines for diagnosis, treatment and follow-up. Ann Oncol..

[8] Hampel P.J., Parikh S.A. (2022). Chronic lymphocytic leukemia treatment algorithm 2022. Blood Cancer J.

[9] Sharma RK, Dash B. (2015).

[10] Sharma R.K. (2015). Charaka Samhita of Agnivesha, Chikitsa Sthana; Rajayakshma Chikitsitam; Chapter 8, Verse 87-88.

[11] Shanmugavel M. (1968). Putru noi Patriya vilakamum Siddha maruthuva arachigalaum (Tamil).

[12] Morrison V.A. (1998). The infectious complications of chronic lymphocytic leukemia. Semin Oncol.

[13] Nucci M., Anaissie E.J., Dignani M.C., Mahfouz T., Wiernik P., Goldman J., Dutcher J., Kyle R. (2013). Neoplastic diseases of the blood.

[14] Stephens J.M., Gramegna P., Laskin B., Botteman M.F., Pashos C.L. (2005). Chronic lymphocytic leukemia: economic burden and quality of life: literature review. Am J Ther.

[15] Fortune EE., Miller MF, Kranzler EC, Ackourey J, Kelly C, Badt H (2020). Financial burden is associated with postponing care and decreasing physical and emotional quality of life among patients with multiple myeloma and chronic lymphocytic leukemia. Blood.

[16] Mathew S., Kuttan G. (1997). Antioxidant activity of Tinospora cordifolia and its usefulness in the amelioration of cyclophosphamide induced toxicity. J Exp Clin Cancer Res.

[17] Mohan V., Ashwani K. (2022). Pro-apoptotic, anti-metastatic, and anti-telomerase activity of *Tinospora cordifolia* and its active polysaccharide arabinogalactan during Benzo(a)pyrene-induced lung carcinogenesis. Indian J Pharmacol.

[18] Kalikar M.V., Thawani V.R., Varadpande U.K., Sontakke S.D., Singh R.P., Khiyani R.K. (2008). Immunomodulatory effect of Tinospora cordifolia extract in human immuno-deficiency virus positive patients. Indian J Pharmacol.

[19] Sharma V., Katiyar A., Agrawal R.C. (2018). Glycyrrhiza glabra: chemistry and pharmacological activity. Sweeteners.

[20] Bisht D, Rashid M, Arya RKK, Kumar D, Chaudhary SK, Rana VS (2022). Revisiting liquorice (Glycyrrhiza glabra L.) as anti-inflammatory, antivirals and immunomodulators: Potential pharmacological applications with mechanistic insight. Phytomed Plus.

[21] Ayeka P.A., Bian Y., Githaiga P.M., Zhao Y. (2017). The immunomodulatory activities of licorice polysaccharides (Glycyrrhiza uralensis Fisch.) in CT 26 tumor-bearing mice. BMC Complement Altern Med.

[22] Hostetler B.J., Uchakina O.N., Ban H., McKallip R.J. (2017). Treatment of hematological malignancies with glycyrrhizic acid. Anticancer Res.

[23] Ngamkitidechakul C., Jaijoy K., Hansakul P., Soonthornchareonnon N., Sireeratawong S. (2010). Antitumour effects of Phyllanthus emblica L.: induction of cancer cell apoptosis and inhibition of in vivo tumour promotion and in vitro invasion of human cancer cells. Phytother Res.

[24] Sugapriya D., Shanthi P., Sachdanandam P. (2008). Restoration of energy metabolism in leukemic mice treated by a siddha drug--Semecarpus anacardium Linn. nut milk extract. Chem Biol Interact.

[25] Kawaii S., Lansky E.P. (2004 Spring). Differentiation-promoting activity of pomegranate (Punica granatum) fruit extracts in HL-60 human promyelocytic leukemia cells. J Med Food.

[26] Tsantila E.M., Esslinger N., Christou M., Papageorgis P., Neophytou C.M. (2024). Antioxidant and anticancer activity of *Vitis vinifera* extracts in breast cell lines. Life.

[27] Turrini E., Calcabrini C., Sestili P., Catanzaro E., de Gianni E., Diaz A.R., Hrelia P., Tacchini M., Guerrini A., Canonico B., Papa S., Valdrè G., Fimognari C. (2016). Withania somnifera induces cytotoxic and cytostatic effects on human T leukemia cells. Toxins.

[28] Haskin G., Kogan M. (2018). Case report of unexpectedly long survival of patient with chronic lymphocytic leukemia: why integrative methods matter. Integr Med.

